# Supplementary material for: Varying Cellular Immune Response against SARS-CoV-2 after the Booster Vaccination: A Cohort Study from Fukushima Vaccination Community Survey, Japan
Source: Vaccines (Basel). 2023 Apr 29;11(5):920. doi: 10.3390/vaccines11050920 (PMC10220831; doi:10.3390/vaccines11050920)
Supplement: Supplementary file 1 [file vaccines-11-00920-s001.zip › vaccines-2352914-supplementary.pdf]

Supplemental Material

Table S1. Descriptive detail data of reactive interpretation of T-SPOT.*COVID*

Table S2. Missing data of reactive interpretation of T-SPOT.*COVID*

Table S3. Details of the information and results of the previously infected participants

Table S4. Descriptive data of reactive interpretation of T-SPOT.*COVID*

Figure S1. Difference in anti-SARS-COV-2 IgG titer between non-reactive & borderline and reactive group of T-SPOT.*COVID*

Table S1. Descriptive detail data of reactive interpretation of T-SPOT.*COVID*.

Variables	N=1089	Reactive Interpretation		
		Non-Reactive (n =250)	Reactive (n =700)	Borderline (n =139)
Comorbidities				
At least one	616 (56.4)	182 (72.8)	355 (50.7)	79 (56.8)
DETAILS				
Hypertension	295 (27.0)	102 (41.0)	152 (21.8)	41 (29.5)
Dyslipidemia	95 (8.7)	22 (8.8)	59 (8.5)	14 (10.1)
Heart disease	96 (8.8)	35 (14.1)	46 (6.6)	15 (10.8)
Diabetes	88 (8.1)	36 (14.5)	42 (6.0)	10 (7.2)
Allergic disease	54 (4.9)	9 (3.6)	39 (5.6)	6 (4.3)
Asthma	43 (3.9)	10 (4.0)	28 (4.0)	5 (3.6)
Liver disease	69 (6.3)	22 (8.8)	38 (5.4)	9 (6.5)
Cancer	42 (3.8)	17 (6.8)	19 (2.7)	6 (4.3)
Gout	23 (2.1)	9 (3.6)	10 (1.4)	4 (2.9)
Thyroid disease	30 (2.7)	5 (2.0)	22 (3.2)	3 (2.2)
Lung disease	23 (2.1)	10 (4.0)	11 (1.6)	2 (1.4)
Mental disease	20 (1.8)	11 (4.4)	6 (0.9)	3 (2.2)
Rheumatism	21 (1.9)	11 (4.4)	7 (1.0)	3 (2.2)
Kidney disease	8 (0.7)	2 (0.8)	4 (0.6)	2 (1.4)
Anaphylaxis	8 (0.7)	3 (1.2)	4 (0.6)	1 (0.7)
Collagen disease	8 (0.7)	3 (1.2)	3 (0.4)	2 (1.4)
Immune deficiency	4 (0.4)	1 (0.4)	2 (0.3)	1 (0.7)
Others	197 (18.0)	68 (27.4)	109 (15.6)	20 (14.4)
Drug				
At least one	180 (16.5)	62 (24.8)	87 (12.4)	31 (22.3)
DETAILS				
Steroid	32 (2.9)	15 (6.1)	14 (2.1)	3 (2.2)
NSAIDs	83 (7.6)	27 (11.0)	43 (6.3)	13 (9.6)
Acetaminophen	34 (3.1)	9 (3.7)	18 (2.6)	7 (5.1)
Antihistamine	50 (4.6)	16 (6.5)	27 (3.9)	7 (5.1)
Biologics	14 (1.3)	5 (2.0)	8 (1.2)	1 (0.7)
Anticancer agent	5 (0.5)	0 (0)	4 (0.6)	1 (0.7)
Immunosuppressants	4 (0.4)	2 (0.8)	2 (0.3)	0 (0)
Adverse reaction after 2nd dose				
At least once	825 (75.5)	137 (54.8)	587 (84.0)	101 (73.2)
DETAILS				
Local Pain	563 (51.6)	88 (35.3)	405 (58.0)	70 (50.7)
Fever over 37.5 degree	154 (14.1)	18 (7.2)	123 (17.6)	13 (9.4)
Fever under 37.5 degree	265 (24.3)	18 (7.2)	217 (31.1)	30 (21.6)
Fatigue	514 (47.1)	59 (23.7)	396 (56.7)	59 (42.4)
Joint pain	273 (25.0)	24 (9.6)	222 (31.8)	27 (19.4)
Headache	317 (29.0)	43 (17.3)	240 (34.4)	34 (24.5)
Dizziness	52 (4.8)	6 (2.4)	45 (6.4)	1 (0.7)
Nausea	46 (4.2)	3 (1.2)	38 (5.4)	5 (3.6)
Diarrhea	27 (2.5)	1 (0.4)	24 (3.4)	2 (1.4)
Others	55 (5.0)	10 (4.0)	36 (5.2)	9 (6.5)
Adverse reaction after 3rd dose				

At least once	876 (80.2)	153 (62.2)	612 (88.3)	111 (81.0)
DETAIL				
Local Pain	663 (60.7)	115 (46.0)	454 (65.2)	94 (67.6)
Fever over 37.5 degree	135 (12.4)	16 (6.4)	97 (13.9)	22 (15.8)
Fever under 37.5 degree	285 (26.1)	16 (6.4)	253 (36.4)	16 (11.5)
Fatigue	474 (43.4)	43 (17.2)	377 (54.2)	54 (38.8)
Joint pain	323 (29.6)	37 (14.8)	251 (36.1)	35 (25.2)
Headache	294 (26.9)	29 (11.6)	239 (34.3)	26 (18.7)
Dizziness	51 (4.7)	7 (2.8)	40 (5.7)	4 (2.9)
Nausea	46 (4.2)	4 (1.6)	36 (5.2)	6 (4.3)
Diarrhea	31 (2.8)	4 (1.6)	25 (3.6)	2 (1.4)
Others	69 (6.3)	5 (2.0)	57 (8.2)	7 (5.0)

Number (percentage) are shown for continuous or categorical variables. Abbreviations; NSAIDs: Non-Steroidal Anti-Inflammatory Drugs.

Table S2. Missing data of reactive interpretation of T-SPOT.*COVID*

Variables	Reactive Interpretation		
	Non-Reactive (n =250)	Reactive (n =700)	Borderline (n =139)
Age	0 (0)	0 (0)	0 (0)
Sex	0 (0)	0 (0)	0 (0)
Blood type	33 (13.2)	27 (3.9)	3 (2.2)
Days between booster vaccination and blood collection	0 (0)	0 (0)	0 (0)
Vaccine type	0 (0)	0 (0)	0 (0)
Previous infection	0 (0)	0 (0)	0 (0)
IgG (S)	0 (0)	0 (0)	0 (0)
Smoking	8 (3.2)	13 (1.9)	4 (2.9)
Drinking Habits	11 (4.4)	20 (2.9)	8 (5.8)
Comorbidities	0 (0)	0 (0)	0 (0)
Drug	0 (0)	0 (0)	0 (0)
Adverse reaction after 2nd and 3rd dose vaccination	4 (1.6)	8 (1.1)	3 (2.2)
Adverse reaction after 2nd dose vaccination	0 (0)	1 (0.1)	1 (0.7)
Adverse reaction after 3rd dose vaccination	4 (1.6)	7 (1.0)	2 (1.4)

Number (percentage) are shown for continuous or categorical variables.

Table S3. Details of the information and results of the previously infected participants

#	sex	age	comorbidity	AR after 2nd / 3rd dose	Days between booster vaccination and blood collection	T-SPOT.*COVID*	Nab (AU/ml)	IgG (S) (AU/ml)
1	Female	21	-	+/+	57	3	>500	9886
2	Female	68	HTN, HD, CD	+/+	60	5	>500	5372
3	Female	42	DM	+/+	26	9	>500	8657
4	Female	51	-	+/+	58	28	>500	5389
5	Female	39	-	+/+	58	50	>500	8853

Median [Inter Quartile] or number (percentage) are shown for continuous or categorical variables. AR adverse reaction, HTN hypertension, HD heart disease CD collagen disease, DM diabetes mellitus. Of note, we observed one participant with a nonreactive T-SPOT.COVID result, one with a borderline result, and three with reactive results. These findings align with the cohort without a history of COVID-19 infection (n =1,084), in which 249 (22.9%) individuals had nonreactive results, 138 (12.7%) had borderline results, and 697 (64.3%) had reactive results. In humoral immune response, all individuals with a history of COVID-19 infection showed strong reactivity of neutralizing antibody titer > 500 AU/ml and anti-SARS-CoV-2 IgG >5,000 AU/mL.

Table S4. Descriptive data of reactive interpretation of T-SPOT.*COVID*

Variables	N=1089	Reactive Interpretation		
		Non-Reactive (n =250)	Reactive (n =700)	Borderline (n =139)
Age	56 [42–68]			
<40 years	215 (19.7)	19 (7.6)	177 (25.3)	19 (13.7)
40–65 years	543 (49.7)	93 (37.2)	388 (55.4)	62 (44.6)
>65 years	331 (30.3)	138 (55.2)	135 (19.3)	58 (41.7)
Sex - Female	737 (67.5)	151 (60.4)	487 (69.6)	99 (71.2)
Blood type				
A	392 (35.9)	87 (40.1)	257 (38.2)	48 (35.3)
B	223 (20.4)	49 (22.6)	139 (20.7)	35 (25.7)
O	316 (28.9)	66 (30.4)	211 (31.4)	39 (28.7)
AB	95 (8.7)	15 (6.9)	66 (9.8)	14 (10.3)
Days from booster vaccination	53 [29–65]	54 [30–65]	52 [29–65]	54 [29–64]
<30 days	332 (30.4)	77 (30.8)	212 (30.3)	43 (30.9)
30–60 days	367 (33.6)	83 (33.2)	237 (33.9)	47 (33.8)
>60 days	390 (35.7)	90 (36.0)	251 (35.9)	49 (35.3)
Vaccine type				
BNT162b2 (Pfizer–BioNTech)	682 (62.5)	131 (52.4)	459 (65.6)	92 (66.2)
mRNA-1273 (Moderna)	407 (37.3)	119 (47.6)	241 (34.4)	47 (33.8)
Previous infection- IgN≧10.0	11 (1.0)	1 (0.0)	9 (0.8)	1 (0.0)
anti-SARS-COV-2 IgG titter (AU/ml)	2221 [1376–3966]	1593 [977.5–2603]	2674 [1608–4418]	1853 [1202–3606]
Smoking	194 (17.8)	38 (15.7)	132 (19.2)	24 (17.8)
Drinking Habits	406 (37.2)	83 (34.7)	276 (40.6)	47 (35.9)
Comorbidities - At least one	616 (56.4)	182 (72.8)	355 (50.7)	79 (56.8)
Drug - At least one	180 (16.5)	62 (24.8)	87 (12.4)	31 (22.3)
Adverse reaction after 2nd and 3rd dose vaccination				
At least once	930 (85.2)	175 (71.1)	636 (91.9)	119 (87.5)
Adverse reaction after 2nd dose vaccination				
At least once	825 (75.5)	137 (54.8)	587 (84.0)	101 (73.2)
Adverse reaction after 3rd dose vaccination				
At least once	876 (80.2)	153 (62.2)	612 (88.3)	111 (81.0)

Median [Inter Quartile] or number (percentage) are shown for continuous or categorical variables. Of note, a significant difference in age groups was observed between those receiving BNT162b2 and mRNA-1273 vaccines for booster administration. (p with Pearson's chi-square test <0.001); the numbers (percentages) in <40 years, 40–65 years, and >65 years old were 150 (22.0), 391 (57.3), and 141 (20.7) in BNT162b2, and 65 (16.0), 152 (37.3), 190 (46.7) in mRNA-1273 group, respectively.

Figure S1. Difference in anti-SARS-COV-2 IgG titer between non-reactive & borderline and reactive group of T-SPOT.*COVID*

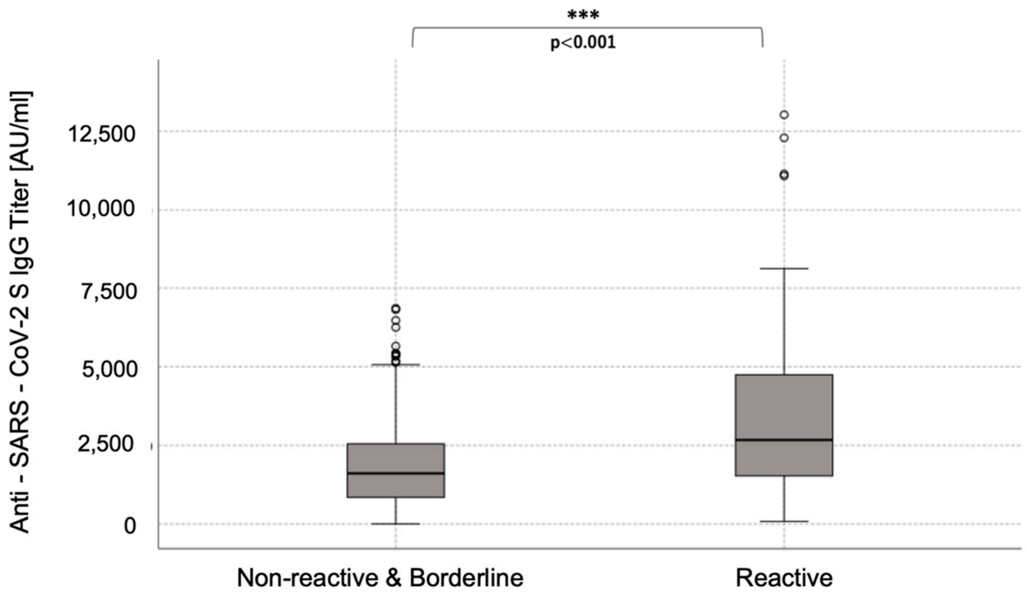

Box-and-whisker plots show the difference in anti-SARS-COV-2 IgG titer between non-reactive & borderline and reactive group of T-SPOT.*COVID*. In each box-and-whisker plot, the horizontal line represents the median, the top and bottom of the boxes the interquartile range, and the I bar the minimum and maximum values. Spots≧50 is scored as 50. Spots ≦4, 5–7, and ≧8 were considered as non-reactive, borderline and reactive respectively.
